# Return to work during coronavirus disease 2019 (COVID-19): Temperature screening is no panacea

**DOI:** 10.1017/ice.2020.1225

**Published:** 2020-09-23

**Authors:** David H. Slade, Michael S. Sinha

**Affiliations:** 1 Department of Infectious Diseases, Loyola University Medical Center and Hines VA Medical Center, Hines, IL; 2 Harvard-MIT Center for Regulatory Science, Harvard Medical School, Boston, Massachusetts; 3 Program on Regulation, therapeutics, and Law (PORTAL), Brigham and Women’s Hospital, Boston, Massachusetts

*To the Editor—*In the midst of the current pandemic, employee screening is a critical component of reopening businesses, but cost is an important consideration.^1^ Screening involves a designated individual asking symptom-related questions and performing a temperature check of employees as they enter the premises. Some state governors have issued executive orders requiring temperature checks, and many large businesses have implemented automated systems for checking temperature, including Amazon and Emirates Airlines. The Centers for Disease Control and Prevention (CDC) recommends that employers implement symptom and temperature screening.^2^ The Centers for Medicare and Medicaid Services (CMS) also requires this screening and will be auditing healthcare facilities to ensure compliance. Failing to comply would place hospitals at risk of losing Medicare funding and incurring major financial losses. Temperature screening for coronavirus disease 2019 (COVID-19) is thought to have little down side, but in practice it does little to prevent the spread of the virus. The cost of paying staff and the oversight required to implement and monitor such a program diverts valuable resources away from more effective measures.

Temperature screening can be performed in several ways: (1) home screening using commercial thermometers; (2) in person temperature measurement with noncontact infrared thermometers; and (3) automated noncontact thermal imaging cameras. Home screening is the most cost-effective option, but in practice employees cannot be relied upon to consistently and accurately measure and self report temperature. For in-person screening, noncontact infrared thermometers can be used at employee entrances, but the close contact required for measurement places both parties at risk of COVID-19 transmission. Measurements are frequently inaccurate due to inaccurate positioning of the thermometer relative to the examinee, and the cost of paying an hourly employee to perform screening is high and not feasible for after-hours access. At first glance, temperature screening seems highly appealing, in that it offers objective data for monitoring employees. Yet when implemented at scale, the cost of temperature screening can quickly escalate to millions of dollars.

To reduce costs associated with hiring employees for screening, thermal imaging cameras may appeal to some as a one-time investment with low cost of maintenance. An employee stands within 1–2 m (3–6 feet) of the device, aligns their face with the camera, and the temperature is registered from either the forehead or tear duct. Tear duct measurement is preferred, as it measures temperature from a single artery.^3^ Thermal imaging cameras provide comparable accuracy to oral temperature readings, but only when routinely calibrated and adjusted to control for individual and environmental factors. The accuracy of such devices can be affected by the presence of facial hair, wigs, eyeglasses, masks, hats, the employee’s height, use of a wheelchair, and other external sources of temperature variation, such as a hot beverage. For those entering from a cold environment, facial skin must reacclimate to ambient temperature,^3^ which may contribute to crowding at the entrance. Moreover, the screening area where thermal imaging cameras are placed must be maintained at relatively consistent ambient temperature and humidity; this may be difficult to achieve in entrances to facilities, particularly in extreme weather conditions. Finally, these devices still require paying employees to monitor them.

Numerous reports have shown that temperature screening rarely identifies elevated temperatures.^4^ For example, at Loyola University Medical Center, where one of us is employed, from the time temperature screening was implemented on May 1 through July 31, several thousand screens have been performed, with zero positive temperature readings. There may be good explanations for this. First, an employee with a subjective fever is more likely to stay home. In practice, given the limitations discussed above, device readings are frequently inaccurate and may miss some low-grade temperatures. Fever is transient and follows a diurnal pattern, making a one-time morning spot check of temperature a poor means of monitoring for fever. Antipyretic agents such as acetaminophen could mask the presence of a fever. Fever is also not present in every patient with COVID-19, and by definition, is not present in an asymptomatic individual. Finally, fever is nonspecific for COVID-19, so elevated temperatures captured by screening could be due to other illnesses such as seasonal influenza.

Recently, the value of temperature screening has been called into question. Dr Anthony Fauci described temperature checks as “notoriously inaccurate,”^5^ and the Administrator of the Transportation Security Administration (TSA) similarly cast doubt upon their reliability.^6^ We recommend abandoning the use of temperature checks for employee screening. On-site temperature screening is a high-cost, low-yield tool for preventing the spread of COVID-19. It will fail to catch most COVID-19 cases, and the expenditure required to support on-site screening may require other budgetary restrictions, such as the furloughing or layoff of employees. Symptom screening alone—combined with strict adherence to universal precautions like masking and eye protection—is a superior strategy to prevent spread of COVID-19 in the workplace. A daily questionnaire is a low-cost measure that can identify symptomatic employees, increase awareness, and promote adherence to infection prevention guidelines. Policymakers should follow the evidence, moving away from temperature screening mandates in favor of practices that are better tailored to mitigate risk of COVID-19 transmission while at work.
